# Self-assembled angiopep-2 modified lipid-poly (hypoxic radiosensitized polyprodrug) nanoparticles delivery TMZ for glioma synergistic TMZ and RT therapy

**DOI:** 10.1080/10717544.2018.1534897

**Published:** 2019-02-11

**Authors:** Zhenkun Zong, Lei Hua, Zhen Wang, Haoyue Xu, Chengkun Ye, Bomin Pan, Zongren Zhao, Longzhen Zhang, Jun Lu, Hongmei Liu, Rutong Yu

**Affiliations:** a Nanjing Medical University, Nanjing, China;; b Institute of Nervous System Diseases, Xuzhou Medical University, Xuzhou, China;; c Department of Neurosurgery, Affiliated Hospital of Xuzhou Medical University, Xuzhou, China;; d Department of Radiation Oncology, Affiliated Hospital of Xuzhou Medical University, Xuzhou, China;; e Key Laboratory for Biotechnology on Medicinal Plants of Jiangsu Province, School of Life Science, Jiangsu Normal University, Xuzhou, China;; f Jiangsu Center for the Collaboration and Innovation of Cancer Biotherapy, Cancer Institute, Xuzhou Medical University, Xuzhou, China

**Keywords:** Glioma, angiopep-2, radiosensitized polyprodrug, TMZ, synergetic therapy

## Abstract

The addition of temozolomide (TMZ) to radiotherapy (RT) improves survival of patients with glioblastoma (GBM). However, TMZ + RT causes excess toxicity in patients. In this study, we prepared angiopep-2 (A2) modified lipid-poly (hypoxic radiosensitized polyprodrug) nanoparticles for TMZ delivery (A2-P(MIs)25/TMZ) to achieve synergistic effects against glioma. This A2-P(MIs)25/TMZ display highly promising advantages: (1) a hydrophobic P-(MIs)25 core where poorly water-soluble TMZ can be encapsulated; (2) nitro groups of the hydrophobic P-(MIs)25 core that are converted into hydrophilic amino groups (P(NH_2_s)25) under low oxygen conditions to mimic the oxygen-increased sensitization to RT; (3) a lipid monolayer at the interface of the core and the shell to modify the A2 (a specific ligand for low-density lipoprotein receptor-related protein-1 (LRP-1), which are expressed in the blood-brain barrier (BBB) and human glioma cells), thereby enhancing the drug encapsulation efficiency in glioma. These nanoparticles appear as a promising and robust nanoplatforms for TMZ and hypoxic cell radiosensitization delivery.

## 1. Introduction

Glioblastoma (GBM), the most common primary brain tumor in adults, is usually rapidly fatal (Sukhdeo et al., 2011). The current standard of care for newly diagnosed GBM is surgical resection to the extent feasible, followed by adjuvant RT and chemotherapy. The median survival of patients with GBM seldom exceeds 14.6 months (Minniti et al., 2008). Glioma is known to be more resistant to radiation, due to its intra-tumoral hypoxia (Zhou et al., 2006; Carlson et al., 2011). Nitroimidazoles as radiosensitizers have been studied in order to increase the sensitivity of hypoxic cells towards radiation, since they are found to mimic the effect of oxygen in the radiochemical process. Metronidazoles (MIs) as nitroimidazole derivatives have been used in controlled trials to evaluate possible enhancement of the radiation effect in patients with malignant glioma (Urtasun et al., 1977; Voronina & Pelevina, 1977; Eyre et al., 1984; Cyb et al., 1985). However, the results of these clinical trials have been not generally satisfactory. The most important factor underlying the failure of MI to achieve a useful advantage is undoubtedly the low plasma concentrations achievable with the permitted dose of this neurotoxic drug (Urtasun et al., 1974; Frytak et al., 1978). However, high-dose MIs and neurological toxic effects limit their clinical applications (Urtasun et al., 1974; 1976). Hence, the major challenge for the use of nitroimidazoles for hypoxic cell radiosensitization is to develop a method to elevate the drug concentration in the tumor and minimize their toxic side effects on normal tissues.

Temozolomide (TMZ), is a pro-drug releasing a DNA alkylating agent that is the most effective drug in patients with GBM (Friedman et al., 2000; Chang et al., 2004; Mason & Cairncross, 2005; Gondi et al., 2015), due to its penetrating blood-brain barrier (BBB). However, TMZ increases the risk of hematologic complications such as thrombocytopenia, and other side effects include nausea, fatigue, and a decreased appetite (Villano et al., 2012). Meanwhile, TMZ, which is rapid clearance and instability under normal physiological conditions, is limited to the efficacy of anti-glioblastoma drugs (Saleem et al., 2003). A series of recently published studies have shown that radiotherapy (RT) plus TMZ may prolong survival in patients with GBM (Stupp et al., 2005; Irwin et al., 2007; Komotar et al., 2008; Minniti et al., 2008; 2012; Lee et al., 2013). However, compared with RT alone or TMZ alone, TMZ combined with radiation to cure GBM increased the severe side effects on patients. Therefore, the development of new approaches to improve radiochemotherapy for glioma treatment and reduce irradiation of normal brain tissue is necessary.

Over the past few years, great efforts have been made to overcome these obstacles. Recently with the development of nanotechnology, nanoparticle therapeutic carriers have provided new opportunities to achieve effective synergistic therapeutic distribution at tumor sites (Stylianopoulos, 2013; Brunetti et al., 2015; Kunjachan et al., 2015; Liu et al., 2016). These drug carriers are passively or actively targeted to tumors through the enhanced permeability and retention (EPR) effect and a ligand binding to a receptor at the cell surface, so they are ideally suited for the delivery of anticancer drugs in cancer treatment. The complexity of glioma, especially the existence of the BBB, the drug delivery systems must deliver drugs across the BBB for glioma therapy.

**Scheme 1.**
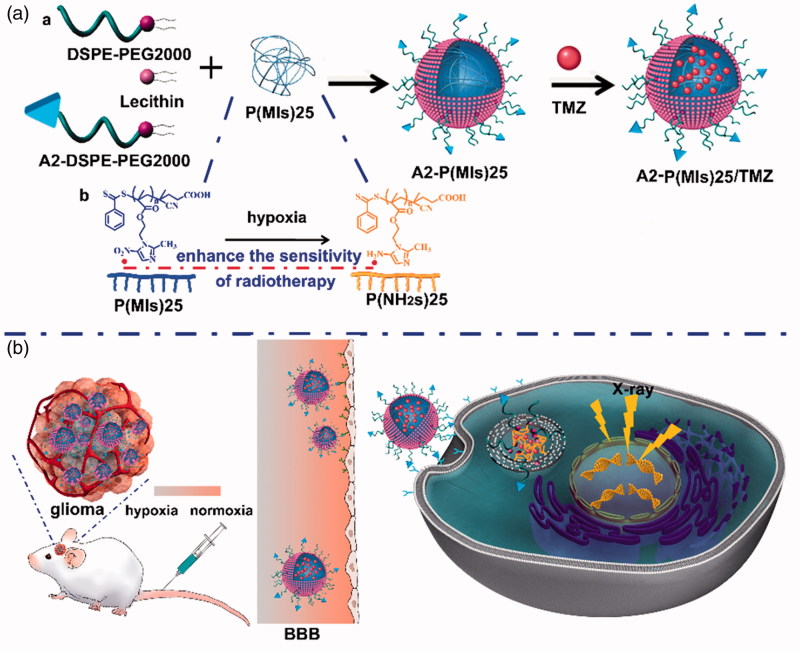
Schematic of A2 modified lipid-poly (hypoxic radiosensitized polyprodrug) nanoparticles delivery TMZ (A2-P(MIs)25/TMZ) for glioma synergetic TMZ and RT therapy. (A) (a) Prepare of A2-P(MIs)25/TMZ; (b) Mechanism of radiosensitization of P(MIs)25; (B) Schematic illustrating A2-P(MIs)25 as hypoxic tumor cell radiosensitizer delivery TMZ to achieve synergistic TMZ and RT treatment of glioma.

In this study, Poly-(MIs)25 (P(MIs)25) as hydrophobic core, which have radiosensitization effect on hypoxic tumors, were synthesized by RAFT polymerization. And then, P(MIs)25 together with TMZ, DSPE-PEG2000, lecithin, and A2-DSPE-PEG2000 were self-assembled into A2-P(MIs)25/TMZ through a single-step, nanoprecipitation step (Scheme 1A(a)). The A2-P(MIs)25/TMZ was mainly comprised of two distinct functional components: (1) a hydrophobic P(MIs)25 core had the following purposes: (i) increased RT sensitivity of hypoxic tumor, and (ii) encapsulated poorly water-soluble TMZ to achieve the synergetic TMZ and RT against glioma; (2) a lipid monolayer at the interface of the core and the shell to modify A2 to enhance glioma targeting and tissue penetration. These features make A2-P(MIs)25/TMZ an excellent carrier for the synergetic TMZ and RT against glioma. In order to confirm the synergetic TMZ and RT of our A2-P(MIs)25/TMZ, A2-PLGA/TMZ without radiosensitization were constructed as the control.

After tail vein injection, A2-P(MIs)25/TMZ was able to penetrate the BBB and enter the glioma tumor due to the EPR effect and active target. It was then internalized into cells by endocytosis. Hypoxia can induce conversion of the hydrophobic P(MIs)25 core to P(NH_2_s)25 through the transfer of six electrons (Edwards, 1993). Due to their electron affinity, MIs increased the radiosensitivity of radio-resistant hypoxic cells, enhancing the DNA damage induced by ionizing radiation (Scheme 1A(b)). The TMZ combined with the P(MIs)25 radiosensitization effects, enhance the synergetic chemo-/radio-therapy against glioma (Scheme 1(B)).

## 2. Materials and methods

### 2.1. Materials

4, 4′-Azobis (4-cyanovaleric acid) (V-501, Aldrich, 99%) was bought from Sigma Aldrich (Saint Louis, MO) and purified by precipitation in methyl alcohol followed by drying under vacuum at 25 °C overnight. The chain-transfer agent (4-cyanopentanoic acid dithiobenzoate, CPADB) for RAFT polymerization were bought from J&K Scientific Ltd (Beijing, China). 1, 2-dioleoylsn-glycero-3-phosphoethanolamine-n-[poly(ethylene glycol)] 2000 (DSPE- PEG2000) were purchased from Shanghai Advance Vehicle Technology Pharmaceutical Ltd (Shanghai, China). DSPE-PEG2000-PDP was purchased from Xian Ruixi Biological Technology Co., Ltd (Xian, China). Angiopep-2 (TFFYGGSRGKRNNF KTEEY) was purchased from GL Biochem Ltd (Shanghai, China). Doxorubicin (DOX), TMZ and D-Luciferin potassium salt were got from Dalian Meilun Biotech Co., Ltd (Dalian, China). Poly-lactic-co-glycolic acid (PLGA), (3-(4,5-Dimethylthiazol-2-yl)-2,5-diphenyltetrazolium bromide (MTT), 4′,6-diamidino-2-phenylindole dihydrochloride (DAPI) were purchased from Sigma Aldrich (Saint Louis, MO). γ-H2AX antibody was obtained from Cell Signaling Technology, Inc. (Danvers, MA). Hypoxyprobe-1 Plus Kit was purchased from hypoxyprobe, Inc. (Burlington, MA). *In Situ* Cell Death Detection Kit was purchased from Roche (Mannheim, Germany).

### 2.2. Nanoparticle preparation and characterization

#### 2.2.1. Preparation of MI-MA

Metronidazole (MI, 8.55 g, 50 mmol), methacrylic acid (MA, 6.45 g, 75 mmol) and 4-dimethylaminopyridine (DMAP, 3.05 g, 25 mmol) were dissolved in 100 mL of dry dichloromethane (DCM) in an oven-dried 250 mL three-necked round-bottom flask attached to a 100 mL slow-addition apparatus. Next, the mixture was stirred under argon flow for 1 h, then dicyclohexylcarbodiimide (DCC, 20.6 g, 100 mmol, dissolved in 50 mL DCM) was added dropwise. The mixture was stirred at 30 °C overnight. The mixture was cooled to room temperature and filtered. The filter cake was washed with 3 × 50.0 mL DCM. Then the filtrate was combined and washed with water, 3 × 50.0 mL, the organic layer was then dried with anhydrous Na_2_SO_4_ and evaporated. The product was then purified by flash column chromatography on silica gel. Eluting with a mixed solvent of PE/EA (v/v = 1/1) to afford MI-MA (10.9 g, yield 91.5%) as a white solid. ^1 ^H NMR (300 MHz, DMSO-*d*6_,_ ppm): δ 8.04 (s, 1H), 5.95 (s, 1H), 5.68 (s, 1H), 4.70 (m, 2H), 4.49 (m, 2H), 2.50 (s, 3H), 1.82 (s, 3H). ^13 ^C NMR (DMSO-*d*6): δ = 14.35, 18.28, 45.03, 63.09, 126.70 133.61, 135.78, 139.03, 151.91, 166.50 ppm. MS (m/z): 240.09 [M + H]**^+^** calcd for C_10_H_13_N_3_O_4_ 239.09, found 239.09.

#### 2.2.2. Preparation of poly-(MIs)25

The polymerization was carried out in a baked Schlenk tube under argon protection. MI-MA (718 mg, 300 mmol), V-501 (4.8 mg, 30 mg), CPADB (16.8 mg, 60 mmol) and DMSO (1 mL) were added to tube. The solution was deoxygenated by three standard freeze pump thaw cycles. Then, the reaction tube was placed into an oil bath preset at 70 °C. After maintained at 70 °C for a predetermined time, the reaction tube was placed into a cooling water bath. The resultant polymer after participation in methanol appeared as orange-red oil, and dried under vacuum at 25 °C for 24 h. MW_NMR_=6000 Da, PDI =1.12, degree polymerization =25.

#### 2.2.3. Preparation of A2-DSPE-PEG2000

DSPE-PEG2000-PDP and A2 with the same mol were added to the DMSO. The reaction mixture was stirred gently at room temperature for 36 h. The released 2-pyridinethione was measured to characterize the obtained product. The resolution was lyophilized to obtain the final product A2-DSPE-PEG2000. The A2-P(MIs)25 NPs size were adjusted based on the total lipid (lecithin: DSPE-PEG2000: A2-DSPE-PEG2000)/P-(MIs)n weight ratio is 26.25%.

#### 2.2.4. Preparation of A2-P(MIs)25 and A2-P(MIs)25/TMZ

DSPE-PEG2000 (0.44 mg), lecithin (0.13 mg), Aangiopep-2-DSPE-PEG2000 (0.06 mg), P(MIs)25 (2.4 mg) were completely solubilized in DMSO. The mixtures were added together and vortexed vigorously for 2 min, and dripped into water solution under vigorous stirring conditions for 20 min followed by gently stirring for 2 h at room temperature. TMZ (0.53 mg) with 15% weight of A2-P(MIs)25/TMZ (3.56 mg). And then, A2-P(MIs)25 and A2-P(MIs)25/TMZ were purified by being dialyzed against PBS (pH 7.4) for 12 h in order to remove the free TMZ and DMSO. The A2-PLGA, A2-PLGA/TMZ, A2-PLGA/DOX, A2-P(MIs)25/DOX were prepared with the similar described above.

#### 2.2.5. TMZ loading and characterization of A2-P(MIs)25/TMZ and A2-PLGA/TMZ

The TMZ were measured using a UV-vis spectrophotometer (Bio Tek Synergy2) at 327 nm. The loading efficiency and content of TMZ were calculated as: loading efficiency (%) = (weight of loaded drug)/(weight of drug in feed) × 100%; loading content (%) = (weight of loaded drug)/(total weight of nanoparticles) × 100%. Hydrodynamic diameter and polydispersity of the nanoparticles were determined by dynamic light scattering (DLS) using a ZEN3600 Zetasizer NanoZS (Malvern Instruments Ltd., MA). The morphology of A2-P(MIs)25 and A2-PLGA were observed by transmission electron microscopy (TEM) (Fei Tencai G2 T12).

### 2.3. Cell lines

Mice glioma cell line C6 were cultured in DMEM supplemented with 10% heat-inactivated fetal bovine serum (FBS). Cell cultures under normoxic conditions (pO2: 21%) were maintained in a humidified incubator at 37 °C in 5% CO_2_ and 95% air. Hypoxic conditions (pO_2_: 2%) were produced by placing cells in a hypoxia incubator (Thermo Scientific HERAcell 150i) in a mixture of 2% O_2_, 5% CO_2_ and 93% N_2_.

#### 2.3.1. The cellular uptake of DOX-embedded nanoparticles

To detect the cellular uptake of TMZ, we treated C6 cells incubated with DOX-embedded nanoparticles (P(MIs)25/DOX and A2-P(MIs)25/DOX) under normoxic conditions for 2 h. We washed the cells with PBS and fixed them in 4% paraformaldehyde prepared in PBS for 10 min. We stained with DAPI for nuclear localization and obtained the images using OLYMPUS TH4-200 fluorescence microscopy. Flow cytometry was also used to study the cellular uptake of nanoparticles *in vitro*. The cells were harvested, and cell uptake determined from DOX fluorescence per cell using a BD FACSCalibur flow cytometer (Bedford, MA) and FlowJo software for analysis.

#### 2.3.2. Colony formation assay

The experimental method was performed as previously described (Yao et al., 2016). In order to estimate the effects of A2-P(MIs)25 and A2-PLGA on the radiosensitivity of glioma cells, C6 cells were seeded in 6-well plates at appropriate cell densities. 24 h later, cells were treated with A2-P(MIs)25 and A2-PLGA at the dose of 0.18 mg mL^−1^ P-(MIs)25 or PLGA per well. After 5 h, cells were irradiated with 0, 2, 4, 6 and 8 Gy, respectively; the dose rate was 0.3 Gy min^−1^. Cell clones were fixed with 4% paraformaldehyde and stained with crystal violet after 14 days. All colonies with exceeding 50 cells were calculated. The plating efficiency was counted as dividing the number of colonies by the number of cells plated. The radiosensitivity was assessed by the sensitization enhancement ratio (SER).

#### 2.3.3. Immunologic techniques for H2AX labeling

C6 cells were seeded at 10 000 cells per well in a 12-well plate for 24 h before treatment. Cells were treated by incubation with A2-P(MIs)25/TMZ and A2-PLGA/TMZ 4 h under hypoxic condition (pO_2_: 2%), followed by irradiation with 2 Gy using the dose rate 0.3 Gy min^−1^. Cells were stained with an anti-γ-H2AX antibody (red) and DAPI (blue) 24 h after RT. Cells were analyzed using fluorescence microscopy and photographed (Olympus, Japan).

### 2.4. Animals

Male ICR mice with 18–20 g weight were purchased from Beijing HFK Bioscience Co., Ltd. (Beijing, China). Glioma-bearing ICR mice were prepared by intracranial injection (striatum, 1.8 mm right lateral to the bregma and 3 mm of depth) of 1 × 10^5^ C6-Luci cells suspended in 5 µL of serum-free media into male ICR mice, as previously described (Li et al., 2017). All mice received care according to the guidelines in the Guide for the Care and Use of Laboratory Animals. All procedures were authorized by Xuzhou Medical University of China Animal Care and Used committee.

#### 2.4.1. *In vivo* distribution of DOX-embedded nanoparticles

The experimental method was performed as previously described (Li et al., 2017; Liu et al., 2017). Glioma model nude mice were first injected with freshly prepared luciferin substrate and imaged with the Xenogen IVIS Spectrum optical imaging device to prove to have similar volume tumors in the brain at the 7th day after implantation in glioma model of nude mice. After that, glioma model ICR mice were injected intravenously with DOX-embedded nanoparticles (P(MIs)25/DOX, and A2-P(MIs)25/DOX through the tail vein at the dose of 3 mg kg^−1^ DOX per animal. Then at 4 h after administration, the mice were sacrificed, and the glioma model brains were excised carefully and visualized under the *in vivo* real-time fluorescence imaging system. The excised glioma model brains were then fixed with 4% paraformaldehyde for 72 h and further dehydrated in sucrose solution. Slices of 20 µm thickness were prepared and stained with DAPI for 10 min at room temperature. The slices were observed under fluorescence microscopy and photographed (Olympus, Japan).

For hypoxic tissue staining, pimonidazole hydrochloride (Hypoxyprobe^TM^-1) as a hypoxic staining probe was used. The slices were treated according to the manufacturer’s instruction and sections immunofluorescence was evaluated by fluorescence microscopy.

#### 2.4.2. *In vivo* anti-glioma efficiency

The experimental method was performed as previously described (Liu et al., 2017). Glioma cells (C6 cells) were transformed with luciferase gene (*C6-GFP-luci*). The real-time fluorescence imaging analysis was used to evaluate the therapeutic efficiency of different types of formulation from 10 to 27 days after tumor implantation. After 10 days of xenograft glioma, ICR mice were randomly divided into 7 groups (*n* = 10) and housed in a controlled temperature room with regular alternating cycles of light and darkness. At days 12, 14 and 16 after implantation, mice received PBS, PBS + RT, A2-PLGA + RT, free TMZ + RT, A2-P(MIs)25 + RT, A2-P(MIs)25/TMZ + RT and A2-PLGA/TMZ through the tail vein containing TMZ (10 mg kg^−1^) and P(MIs)25, PLGA (47.2 mg kg^−1^) per dose with 2 Gy RT per dose. Radiation treatment was performed by using X-ray (0.3 Gy min^−1^) with whole brain RT and a shield designed to protect the animal’s body. The mice were injected with 15 mg kg^−1^ of freshly prepared luciferin substrate suspended in phosphate-buffered saline (PBS). The mice were anesthetized with 4% chloral hydrate anesthesia and imaged using the IVIS kinetic imaging system (Caliper Life Sciences, Hopkinton, MA). The images were acquired at 15 min after the intraperitoneal injection of luciferin. Tumor growth was monitored by live bioluminescence imaging with the Xenogen IVIS Spectrum optical imaging device (Caliper Life Sciences) at different time points. The bioluminescence signals were analyzed using the Living Image Software (Caliper Life Sciences). The relative tumor inhibition rate was calculated using the following formulas: The relative tumor inhibition rate = the tumor bioluminescence intensity of 27 days/the tumor bioluminescence intensity of 10 days.

Throughout the study, mice were weighed regularly. The survival time was calculated from the day of C6 cell inoculation (0 days) to the day of death. Kaplan–Meier survival curves were plotted for each group. The statistic difference was analyzed using *t*-test where *p* value of <.05 is considered significant.

#### 2.4.3. Toxicity evaluation

C6-bearing ICR mice received injections of PBS, PBS + RT, A2-PLGA + RT, free TMZ + RT, A2-P(MIs)25 + RT, A2-P(MIs)25/TMZ + RT and A2-PLGA/TMZ through the tail vein containing TMZ (10 mg kg^−1^) and P-(MIs)n, PLGA (47.2 mg kg^−1^) per dose with 2 Gy RT per dose on days 12, 14 and 16. One day after the last treatment, two mice of per group were sacrificed, and sections of the main organs (brain, heart, liver, spleen, lung, kidneys) were stained with hematoxylin and eosin.

#### 2.4.4. Immunohistology

The brain sections containing the tumor were incubated with 0.3% Triton X-100 followed by 10% goat serum and were then incubated overnight with TdT-dependent dUTP-biotin nick end labeling (TUNEL) primary antibody at 4 °C. To visualize the TUNEL-positive cells, the sections were incubated with Alexa-594-conjugated secondary antibody for 1 h at room temperature in the dark. DAPI was used to stain the cell nuclei. All sections were examined and photographed with an Olympus IX-71 inverted microscope (Tokyo, Japan). 

### 2.5. Statistical analysis

Statistical significance was analyzed using one-way ANOVA. The experimental results were given in the format of mean, or mean ± SD in the Figures (*p < .05, **p < .01).

## 3. Results and discussion

### 3.1. Synthesis and characterization of P(MIs)25 and A2-DSPE-PEG2000

**Figure 1.**
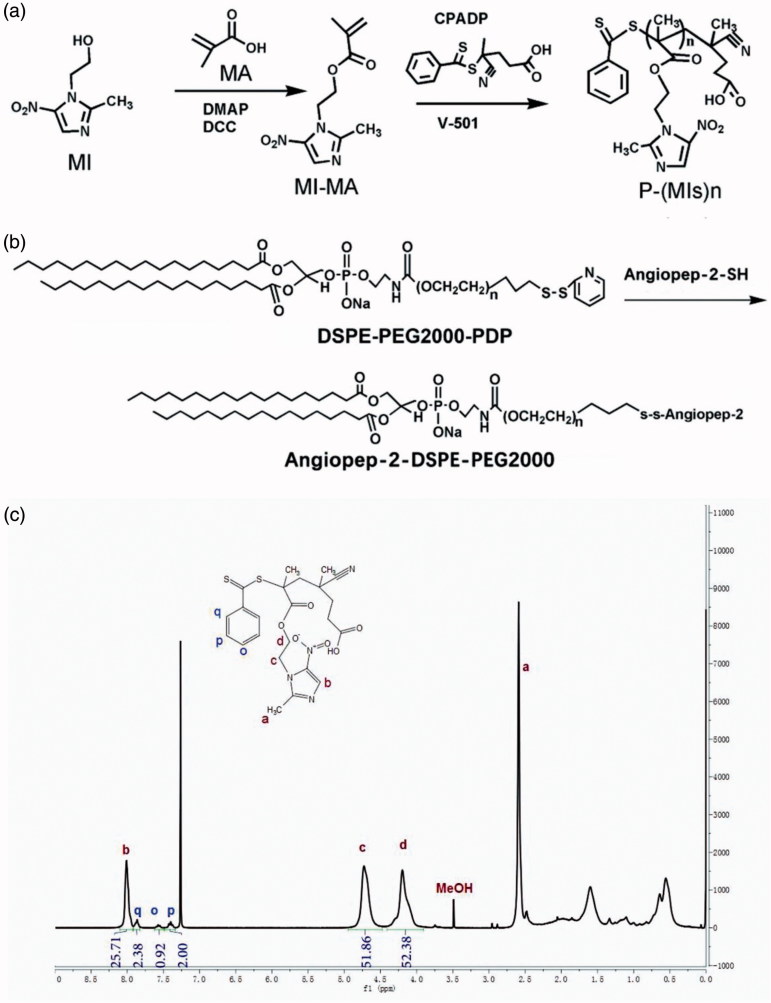
Synthetic routes of (a) P(MIs)n; (b) Angiopep-2-DSPE-PEG2000. (c) ^1^HNMR spectra of P(MIs)25. They were solubilized in CDCl_3_ for ^1^HNMR analysis (300 MHz).

In this study, the P(MIs)25 were prepared according to the steps shown in Figure 1(a). First, a MI-MA was synthesized. The formation of MI-MA was confirmed by ^1 ^H NMR and ^13 ^C NMR spectroscopy and showed all the characteristic peaks and integration values of MI-MA, as seen in Figure S1. The resultant MI-MA was further examined with high-resolution mass spectroscopy for the determination of its mass and molecular formula. The result was consistent with the expected formula of MI-MA (Figure S1). The mass and molecular formula of MI-MA were determine to be MS (m/z): 240.09 [M ＋ H]^+^; the mass calculated for C_10_H_13_N_3_O_4_ was 239.09. These data verified that MI-MA had been synthesized. Second, P-(MIs)25 was prepared by RAFT polymerization. P(MIs)25 were obtained. The ^1 ^H NMR spectra fully confirmed the chemical structures, and the polymerization degree (DP) of P(MIs)25 was calculated from ^1 ^H NMR end group analysis (Figure 1(c)). Moreover, GPC results showed that P(MIs)25 had a narrow distribution of molecular weight with a PDI of 1.12 (Table 1 and Figure S2). DSPE-PEG2000-PDP was chemically modified with A2 through thiol-disulfide exchange (Figure 1(b)) (Lee et al., 2011). The absorbance peak at 343 nm, assigned to the released 2-pyridinethione, indicated that A2 had conjugated to DSPE-PEG2000-PDP to form A2-DSPE-PEG2000. There was no change in the absorbance peak at 343 nm after 30 h, suggesting that the thiol exchange reaction was almost finished (Figure S3) (Liu et al., 2014).

**Table 1.** Molecular characteristics of P-(MIs)25.

Polymer	*M*_n_	*M*_n_^a^	PDI^b^
	(design)	(^1^H NMR)	(GPC)
P-(MIs)25	6000	6200	1.12

*Mn and Mw/Mn* were determined by GPC measurements in DMF (0.35 mL min^−1^, 40 °C, and polystyrene standards).

^a^Determined by ^1^H NMR. ^b^Determined by GPC.

### 3.2. Fabrication and characterization of A2-P(MIs)25/TMZ

**Figure 2.**
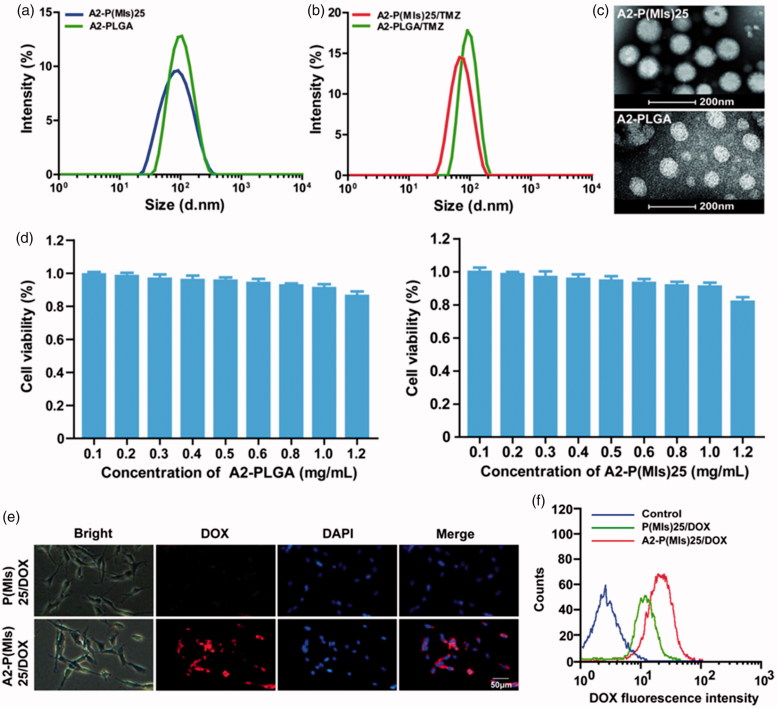
(a) Size distribution of A2-PLGA and A2-P(MIs)25. (b) Size distribution of A2-PLGA/TMZ, and A2-P(MIs)25/TMZ. (c) TEM images of A2-P(MIs)25 and A2-PLGA. (d) The viability of C6 cells cultured with different concentration of A2-PLGA and A2-P(MIs)25 for 48 h by MTT assay. (e) Intracellular release of DOX from P(MIs)25/DOX and A2-P(MIs)25/DOX. Samples were incubated with C6 cells treated with P(MIs)25/DOX and A2-P (MIs)25/DOX for 2 h. Scale bar: 50 μm. (f) Cellular uptake of P(MIs)25/DOX and A2-P(MIs)25/DOX was analyzed by flow cytometry after a 2 h incubation.

After their successful synthesis, P(MIs)25, DSPE-PEG2000, A2-DSPE-PEG2000, TMZ and lecithin were allowed to self-assemble into A2-P(MIs)25/TMZ through a single-step nanoprecipitation step (Zhang et al., 2008; Chan et al., 2009). For comparison, we used a control PLGA as a hydrophobic core to form A2-PLGA/TMZ, due to PLGA without radiosensitization effects on hypoxic tumor cells. To achieve the optimal A2-P(MIs)25 pharmacokinetic properties *in vivo*, the nanoparticle size (diameter, nm) was adjusted based on the total lipid (lecithin: DSPE-PEG2000: angiopep-2-DSPE-PEG2000)/P(MIs)25 weight ratio of 1/3, according to previous reports (Zhang et al., 2008; Chan et al., 2011). Dynamic light scattering (DLS) indicated that the average diameters of A2-P(MIs)25 and A2-PLGA were approximately 74.69 ± 5.73 nm, and 97.24 ± 4.06 nm (Figure 2(a)). The average diameters of A2-P(MIs)25/TMZ, and A2-PLGA/TMZ were similar to those of A2-P(MIs)25 and A2-PLGA at 65.63 ± 4.80 nm, and 93.87 ± 2.45 nm (Figure 2(b)). We further confirmed the morphology of A2-P(MIs)25 and A2-PLGA using TEM. The TEM images suggested that the A2-P(MIs)25 and A2-PLGA were spherical NPs (Figure 2(c)). The TMZ loading efficiency of A2-P(MIs)25/TMZ, and A2-PLGA/TMZ was ∼10.23%, and the MIs loading efficiency of ALP-(MIs)25/TMZ was about 68.18%. Cytotoxicity of A2-P(MIs)25 and A2-PLGA was evaluated in C6 cells using an MTT assay. The viability of the cells was greater than 90% with A2-P(MIs)25 and A2-PLGA concentrations up to 1000 μg mL^−1^, demonstrating a fairly low cytotoxicity of these nanoparticles (Figure 2(d)).

### 3.3. The cellular uptake of nanoparticles

For glioma therapy, selective uptake by glioma cells is an important factor that affects the ultimate therapeutic efficacy. A2, a specific ligand for low-density lipoprotein receptor-related protein-1 (LRP-1) that is expressed in the BBB and human glioma cells, was modified on the surface of P(MIs)25 to enhance the glioma distribution of NPs. A2-conjugated DSPE-PEG2000 was used to decorate the P(MIs)25 to enhance the glioma-targeting ability (denoted as A2-P(MIs)25). To evaluate the glioma-targeting ability of the A2-P(MIs)25/TMZ, fluorescence microscope was used to observe the intracellular distribution of the NPs in C6 glioma cells after 2 h incubation. TMZ itself does not have fluorescence. Thus, in this study, we used DOX as a fluorescent dye to test the ability of cellular uptake by by A2-P(MIs)25/TMZ, due to the similar hydrophobic characteristic of TMZ and DOX. P(MIs)25 and A2-P(MIs)25 were embedded DOX, which presents red fluorescence distribution in cells. C6 cells were incubated with for 2 h. As shown in Figure 2(e), the red fluorescence intensities in the A2-P(MIs)25/DOX-treated group clearly increased, in comparison with that in P(MIs)25/DOX group. To verify the above results, C6 cells were cultured with P(MIs)25/DOX and A2-P(MIs)25/DOX for 2 h before being collected and measured by flow cytometry. The red fluorescence intensities clearly increased with the A2-modified P(MIs)25/DOX (Figure 2(f)). These results reflected a good cellular uptake of TMZ in C6 cells.

### 3.4. The radiosensitizer of A2-P(MIs)25 *in vitro*

**Figure 3.**
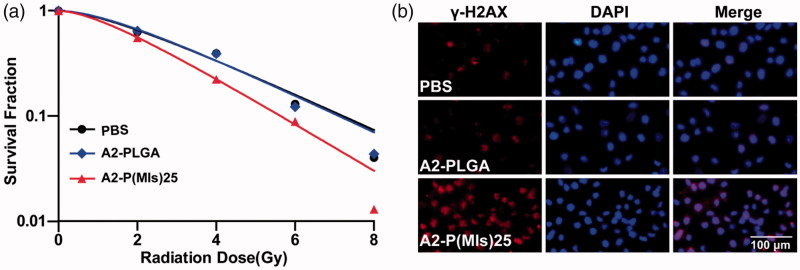
The radiosensitizer of A2-P(MIs)25 *in vitro*. (a) Clonogenic survival curves of C6 cells cultured with A2-PLGA, and A2-P(MIs)25 under hypoxic condition (pO2: 2%) following treatment with 0, 2, 4, 6 and 8 Gy. (b) Immunocytochemical analysis of γ-H2AX expressed C6 cells. Cells were treated by incubation with A2-PLGA, and A2-P(MIs)25 for 4 h under hypoxic condition (pO2: 2%) followed by irradiation with 2 Gy using the dose rate 0.3 Gy min^−1^. Cells were stained with an anti-γ-H2AX antibody (red) and DAPI (blue) 24 h after RT. Scale bar, 100 μm.

Hypoxia can induce conversion of the hydrophobic P-(MIs)25 core to hydrophilic Poly-aminoimidazoles through the transfer of six electrons. Due to their electron affinity, MIs increased the radiosensitivity of radio-resistant hypoxic cells, enhancing the DNA damage induced by ionizing radiation. DNA damage arising from ionization radiation triggers cell apoptosis if DNA repair proteins fail to repair the damage (Thoms & Bristow, 2010). To test whether A2-P(MIs)25 could increase the RT sensitivity of hypoxic tumor cells, a colony-forming assay and immunofluorescence of phospho-Histone H2AX were conducted as the standard method to evaluate the RT-induced apoptosis. As observed for the cell survival in response to ionizing radiation depicted in Figure 3(a), A2-P(MIs)25 was observed to have a RT sensitization function in C6 cells by the downward shift of the survival curves following the administration of both A2-P(MIs)25 and X-rays. Whereas, A2-PLGA under hypoxia showed no obvious radiosensitizing effect on C6 cells. SER of A2-P(MIs)25 and A2-PLGA in C6 cells were 1.31 and 1.04. The degree of DNA damage caused by RT can be assessed by γ-H2AX immunohistochemistry staining, a biomarker for DSBs (Kinner et al., 2008; Mariotti et al., 2013; Zhang et al., 2016). Compared with A2-PLGA under hypoxic conditions, the administration of A2-P(MIs)25 in conjunction with RT significantly increased γ-H2AX under hypoxic conditions because A2-P(MIs)25 acted as hypoxic radiosensitizers to enhance DNA damage caused by radiation (Figure 3(b)). These results demonstrate that A2-P(MIs)25 could effectively enhance the therapeutic X-ray effects and were potent radiosensitizers.

### 3.5. Tumor-therapy monitoring in mice

**Figure 4.**
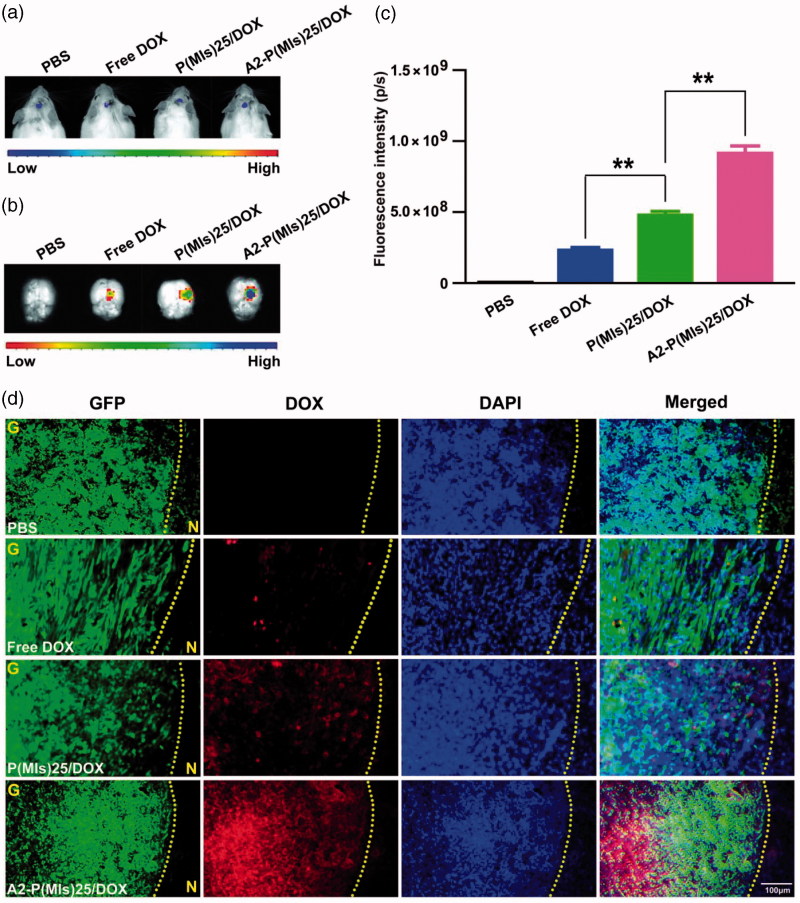
DOX glioma distribution after intravenous injection of mice with either free DOX, P(MIs)25/DOX, and A2-P(MIs)25/DOX. (a) Bioluminescence of luciferase-expressing tumor cells 10 min after injection with luciferin solution. (b) Fluorescence image of free DOX, P(MIs)25/DOX, and A2-P(MIs)25/DOX in excised mouse brains. (c) Quantitative analysis of DOX in excised mouse brains. Data are presented as the mean ± SD (*n* = 3, **p < .01). (d) Fluorescence microscope images showing the distribution of DOX in the glioma following intravenous injection of free DOX, P(MIs)25/DOX, and A2-P(MIs)25/DOX. Green regions were glioma tissues, red regions were DOX fluorescence and cell nuclei were stained with DAPI (blue). Scale bar, 100 µm. G: glioma, N: normal brain tissues. Yellow dashed line = boundary of the glioma.

We continue the *in vivo* experiments due to their beneficial effects for targeting glioma. For glioma-therapy monitoring, orthotopic mouse models of C6-Luci were developed in ICR mice. It was anticipated that A2-P(MIs)25/TMZ could enter the glioma through the EPR effect and active target due to the nanosize and a-2 modification of A2-P(MIs)25/TMZ. To evaluate the glioma targeting properties of A2-P(MIs)25/TMZ, we applied an *in vivo* fluorescence imaging technique to examine the distribution of DOX-labeled nanoparticles after intravenous injection into an orthotopic implantation model of C6-GFP-Luci glioma cells in ICR mice for 4 h. As shown in Figure 4(a), at 7 days after orthotopic implantation of C6-GFP-Luci glioma cells in ICR mice, the luciferase signal was detected and confirmed the presence of brain glioma with almost the same volume. Free DOX, P(MIs)25/DOX, A2-P(MIs)25/DOX and A2-PLGA/DOX were then injected into the tail vein for 4 h and then were carefully excised and visualized using an *in vivo* real-time fluorescence imaging system. As shown in Figure 4(b,c), compared with free DOX and P(MIs)25/DOX, the strongest red fluorescence was observed in gliomas of the mice that had received A2-P(MIs)25/DOX (*p* < .01), indicating an accumulation of A2-P(MIs)25/DOX in the brain tumor due to the A2 effect. Moreover, almost no obvious fluorescence was observed in animals that received an injection of free DOX into their bloodstream because it was rapidly filtered and excreted through the kidneys into the urine. Therefore, free DOX showed less tumor accumulation than P(MIs)25/DOX and A2-P(MIs)25/DOX. Confirmation of the tumor distribution of free DOX, P(MIs)25/DOX, and A2-P(MIs)25/DOX was observed in frozen tumor tissue sections using fluorescence microscopy (Figure 4(d)). The increased red fluorescence in C6-Luci-bearing ICR mice that were injected with A2-P(MIs)25/DOX in tumor tissues effectively demonstrated that A2-P(MIs)25/TMZ was also able to cross the BBB and enter the glioma.

In the above experiment, A2-P(MIs)25/DOX was demonstrated to sensitize glioma cells to RT under hypoxic conditions. The ability of A2-P(MIs)25/DOX to reach hypoxic regions in glioma tumors is essential to allow providers to optimize anti-tumor regimens. The A2-P(MIs)25/DOX distribution in C6 orthotopic transplantation was assessed using an immunofluorescence assay. The hypoxic tissues that were stained with a FITC-labeled monoclonal antibody were found to contain a large amount of embedded A2-P(MIs)25/DOX (Figure S4). A2-P(MIs)25/DOX was thus able to effectively reach the hypoxic tumor site after systemic *in vivo* administration, validating the feasibility of the *in vivo* anti-glioma efficacy test by combining RT with A2-P(MIs)25/TMZ.

**Figure 5.**
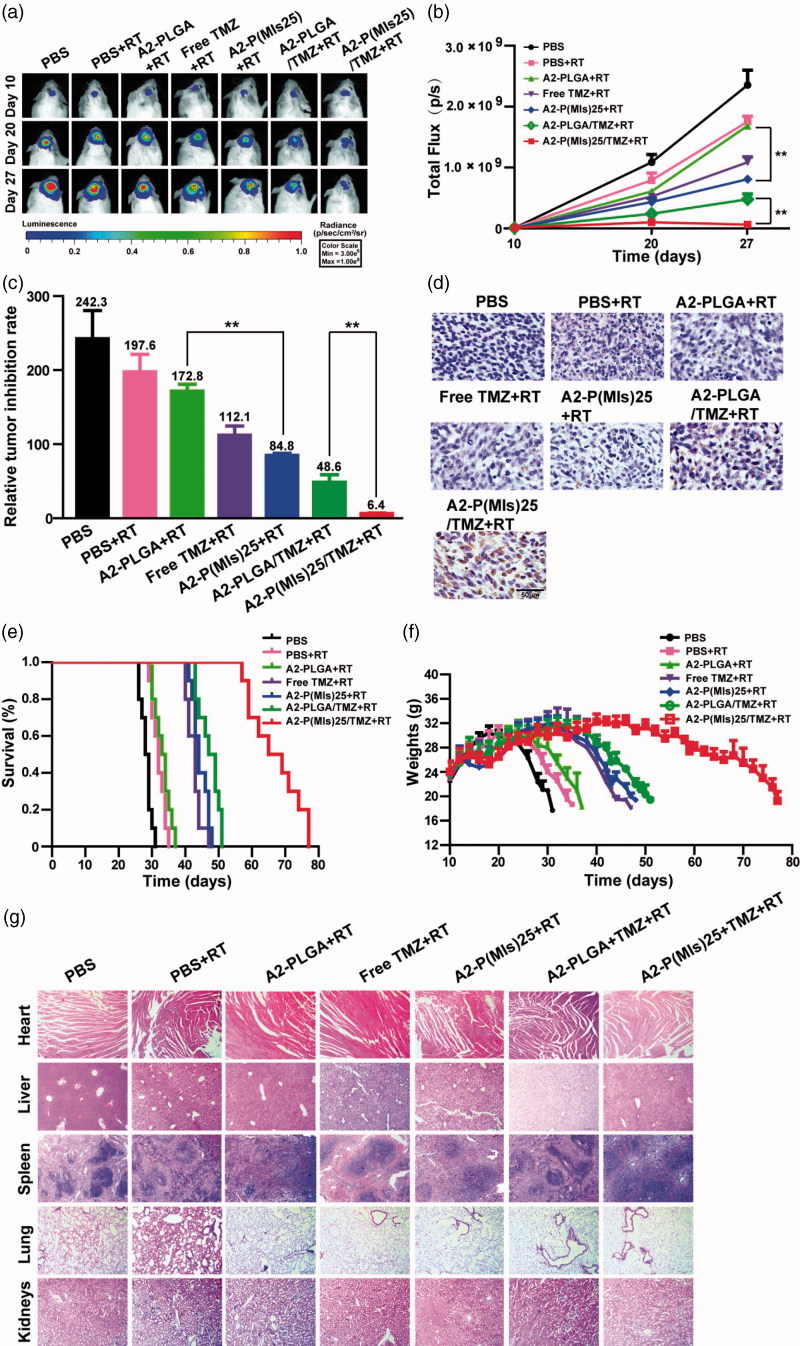
*In vivo* efficacy in the C6-Luci glioma mouse model. (a) C6-Luci-bearing mice received three injections of PBS, PBS + RT, free TMZ + RT, A2-PLGA + RT, A2-P(MIs)25 + RT, A2-PLGA/TMZ + RT group, and A2-P(MIs)25/TMZ + RT at a dose of 10 mg kg^−1^ TMZ and 47.2 mg kg^−1^ P-(MIs) on days 12, 14, and 16, with 2 Gy RT. Bioluminescent signal change correlating to tumor growth over time following inoculation. (b) Quantification of the tumor bioluminescence signal (*n* = 5 mice per group). (c) The tumor growth inhibition results are expressed as mean values with a tumor bioluminescence sensitivity of 27 days to the tumor bioluminescence intensity of 10 days. (d) TUNEL staining of coronal sections from mouse brains with orthotopic tumors. Scale bar, 50 µm. (e) Kaplan–Meier survival curve for the mice (*n* = 10). (f) Body weight change. Data are presented as the mean ± SD (*n* = 10, **p < .01). (g) Histopathological examination of major organs collected after treatment on the animals. H&E staining of major organs. No noticeable abnormality was found in the heart, liver, spleen, lung, or kidneys. Scale bar, 200 µm.

Finally, the anti-glioma efficacy of A2-P(MIs)25/TMZ was estimated in C6-Luci-bearing ICR mice (Figure 5(a)). C6 cells were modified to express the luciferase enzyme, and bioluminescence imaging was used to measure *in vivo* growth. C6-Luci-bearing ICR mice were divided into seven groups (*n* = 10): mice treated with PBS without RT, mice treated with PBS with RT, mice treated with AL-PLGA with RT, mice treated with free TMZ with RT, mice treated with A2-P(MIs)25 with RT, mice treated with A2-PLGA/TMZ with RT, and mice treated with A2-P(MIs)25/TMZ with RT after confirming the presence of brain glioma at 10 days by *in vivo* imaging. As shown in Figure 5(b,c), different formulations with RT had significant effects on inhibiting glioma growth compared with the PBS group (glioma inhibition rate of 242.3) as a negative control. In contrast, due to the polymer PLGA without a radiosensitivity effect on the tumor, the glioma inhibition rate for the A2-PLGA + RT group (172.8), which had almost the same effects on inhibiting glioma growth as the PBS + RT group (197.6). The glioma inhibition rates of A2-P(MIs)25 (84.8) presented a remarkably higher inhibition efficacy towards glioma growth than the PBS + RT group and the A2-PLGA + RT group (*p <* .01), which suggested that P(MIs)25 possessed a radiosensitizing effect. The glioma inhibition rates in the A2-PLGA/TMZ + RT, and A2-P(MIs)25/TMZ + RT groups were 48.6, and 6.4. Moreover, compared with the glioma inhibition rates determined for A2-P(MIs)25+ RT (84.8), and A2-PLGA/TMZ (48.6)+RT, A2-P(MIs)25/TMZ + RT groups clearly demonstrated the strongest inhibition of glioma growth (*p <* .01), suggesting that the combination of A2-P(MIs)25/TMZ enhanced the inhibition of glioma growth. To further evaluate the anti-glioma effects of these NPs on tumor growth, TUNEL were used to show cell apoptosis in xenografts. We also observed an enhancement of apoptosis in P(MIs)25/TMZ + RT, and A2-P(MIs)25/TMZ + RT-treated mice (Figure 5(d)). Collectively, these results suggested that (1) A2-P(MIs)25 + RT effectively enhanced the therapeutic X-ray effects and were potent radiosensitizers, and (2) the combination of TMZ, hypoxic RT sensitization (P(MIs)25) and RT in A2-P(MIs)25/TMZ + RT -treated mice effectively inhibited glioma growth.

To further estimate the antitumor efficacy, the C6-Luci-bearing ICR mice were monitored for body weight changes and median survival times. As shown in Figure 5(e), the median survival times for mice treated with PBS, PBS + RT, A2-PLGA + RT, Free TMZ + RT, A2-P(MIs)25 + RT, A2-PLGA/TMZ + RT, and A2-P(MIs)25/TMZ + RT were 28.5, 32.0, 33.5, 43.0, 44.0, 48.0 and 67.0 days, respectively. The median survival times for the mice treated with A2-P(MIs)25 + RT were longer than those of PBS, PBS + RT, and A2-PLGA + RT, suggesting that A2-P(MIs)25 improved the efficacy of RT. The group that received A2-P(MIs)25/TMZ + RT displayed the longest survival time among all the groups, with high statistical significance compared with the A2-P(MIs)25 + RT, and A2-PLGA/TMZ + RT groups, indicating that the combination of chemotherapy and RT enhanced inhibition of glioma growth. The dominance of A2-P(MIs)25 + RT, and A2-P(MIs)25/TMZ + RT was also reflected by comparing changes in body weight. The body weight of mice treated with A2-P(MIs)25 + RT, A2-P(MIs)25/TMZ + RT slowly decreased, while all other groups lost weight rapidly (Figure 5(f)). Collectively, our results verified that A2-P(MIs)25 was potent radiosensitizers and drug carriers for the delivery of hydrophobic chemotherapy, achieving a synergistic effect with chemoradiotherapy.

Tissue-specific toxicity was examined by histological analysis of various tissues (heart, liver, spleen, lung or kidneys). As shown in Figure 5(g), H&E-stained images of major organs revealed no noticeable tissue damage or obvious changes in morphology in any of the organs in the A2-(MIs)25, A2-P(MIs)25/TMZ groups, as compared with the PBS group. The *in vivo* results suggested that the A2-P(MIs)25 and A2-P(MIs)25/TMZ groups were biocompatible and had potentially positive biological applications with few side effects.

## 4. Conclusions

In summary, A-2 modified lipid-poly (hypoxic radiosensitized polyprodrug) nanoparticles were developed to deliver TMZ (A2-P(MIs)25/TMZ) for glioma synergetic TMZ and RT therapy. The *in vitro* and *in vivo* results demonstrate that these A2-P(MIs)25/TMZ can efficiently target glioma to increase the concentration of TMZ and MIs (hypoxic cell radiosensitizers). The therapeutic studies show that A2-P(MIs)25/TMZ can effectively inhibit the growth of glioma and significantly improve mice survival time without causing obvious adverse effects. Therefore, our A2-P(MIs)25/TMZ can provide a powerful new strategy for enhancing the RT sensitivity of glioma and achieving the synergistic combination of radiation and TMZ for glioma.

## Supplementary Material

Supplementary Figures S1-S4
