# Supplementary material for: Self-assembled angiopep-2 modified lipid-poly (hypoxic radiosensitized polyprodrug) nanoparticles delivery TMZ for glioma synergistic TMZ and RT therapy
Source: Drug Deliv. 2019 Feb 11;26(1):34–44. doi: 10.1080/10717544.2018.1534897 (PMC6394306; doi:10.1080/10717544.2018.1534897)
Supplement: Supplementary Figures S1-S4 [file IDRD_A_1534897_SM2786.docx]

**Supporting Information**
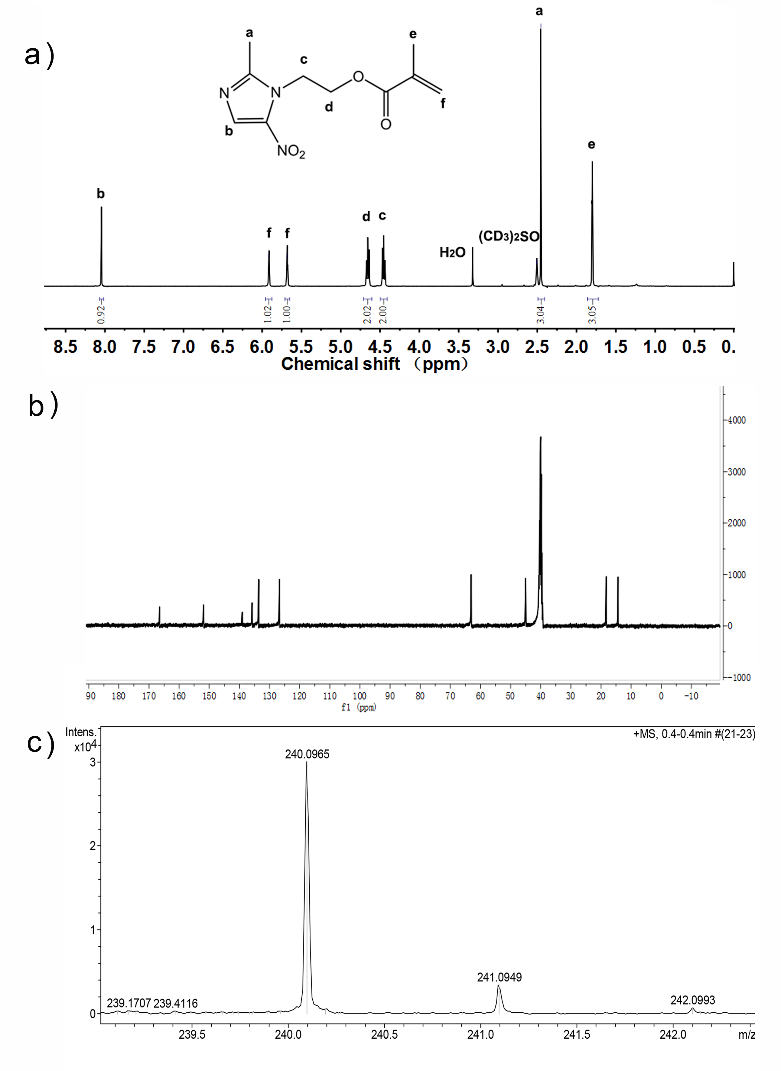


**Figure S1.** a) ^1^H-NMR spectra of MI-MA. They were solubilized in DMSO-*d*6 for ^1^H-NMR analysis (300 MHz). b)^13^C NMR spectra of MI-MA. c) The synthesis of MI-MA was further examined by high-resolution mass spectroscopy.


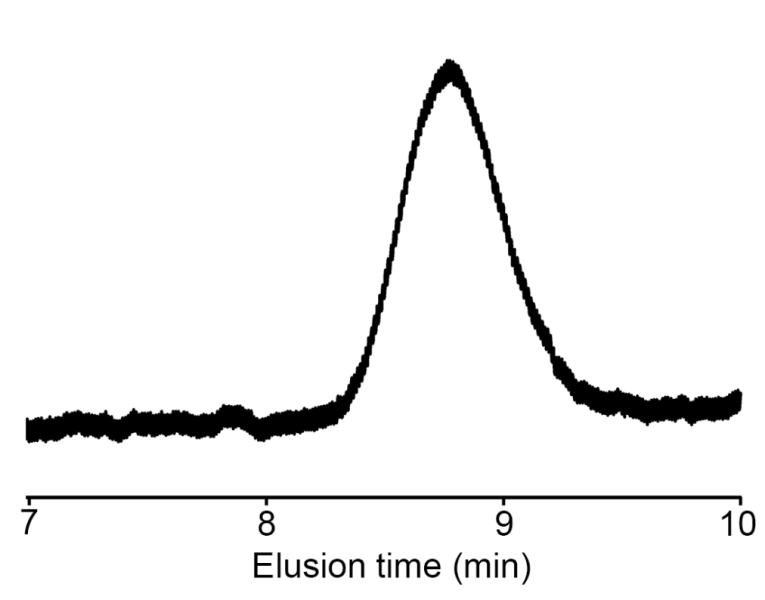


**Figure S2.** GPC profile of A2-P(MIs)25.


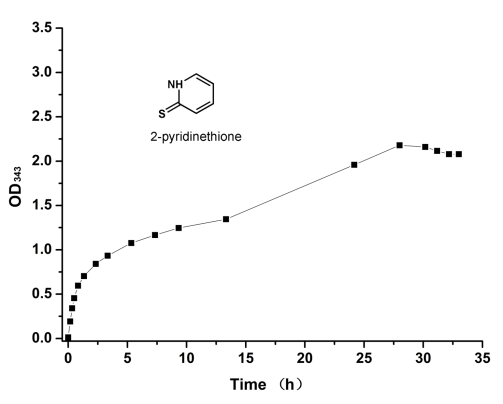


**Figure S3.** The extent of thiol exchange reaction of apopeptide-2-DSPE-PEG2000 conjugates with time measured by UV-vis spectroscopy at 343 nm.


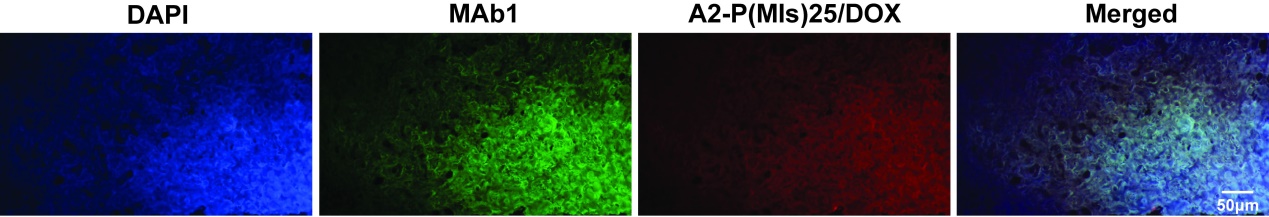


**Figure S4.** Immunofluorescence assay after MAb1 (HypoxyprobeTM-1 Kit), which could bind to proteins, peptides and amino acid adducts of 2-nitroimidazole in hypoxic cells, in addition to tumor tissue sections: green regions are hypoxic tissues, and red regions are DOX fluorescence of A2-P(MIs)25/DOX. Compared with normal brain tissues, glioma cells showed abnormal proliferation, and thus C6-bearing brain tumor tissue was identified based on areas of hypercellularity, as evidenced by the DAPI-stained cell nuclei (blue). Scale bar, 50 µm.
